# Unusual Foreign Bodies in the Orofacial Region

**DOI:** 10.1155/2012/191873

**Published:** 2012-07-09

**Authors:** Sidhi Passi, Neeraj Sharma

**Affiliations:** ^1^Department of Pedodontics, Dr. HSJ Institute of Dental Sciences and Research Center, PU Dental College, Sector 25, Chandigarh, India; ^2^Department of Oral Medicine and Radiology, Dr. HSJ Institue of Dental Sciences and Research, Chandigarh, India

## Abstract

Foreign bodies may be deposited in the oral cavity either by traumatic injury or iatrogenically. Among the commonly encountered iatrogenic foreign bodies are restorative materials like amalgam, obturation materials, broken instruments, needles, and so forth. The discovery of foreign bodies in the teeth is a special situation, which is often diagnosed accidentally. Detailed case history, clinical and radiographic examinations are necessary to come to a conclusion about the nature, size, location of the foreign body, and the difficulty involved in its retrieval. It is more common to find this situation in children as it is a well-known fact that children often tend to have the habit of placing foreign objects in the mouth. Sometimes the foreign objects get stuck in the root canals of the teeth, which the children do not reveal to their parents due to fear. These foreign objects may act as a potential source of infection and may later lead to a painful condition. This paper discusses the presence of unusual foreign bodies—a tip of the metallic compass, stapler pin, copper strip, and a broken sewing needle impregnated in the gingiva and their management.

## 1. Introduction

Self-inflicted injuries are not uncommon [1, 2] and range in severity from simple nail biting to more extreme forms of mutilation, with oral trauma sometimes being the only presenting manifestation. Although the typical clinical features of oral self-injurious behaviour are well documented [3–5], they often present a difficult diagnostic problem for the clinician and, even when recognized, the method of their development and their management are not clearly understood. Foreign bodies may be ingested, inserted into a body cavity, or deposited into the body by a traumatic or iatrogenic injury. Most foreign bodies cause abscess formation, septicemia, or lead to severe haemorrhage; they can also undergo distant embolization [6]. Foreign bodies and tissue reactions to foreign materials, are commonly encountered in the oral cavity. The more common iatrogenic lesions include apical deposition of endodontic materials, mucosal amalgam and graphite tattoos, myospherulosis, oil granulomas, and traumatically introduced dental materials and instruments [7]. Injury to both the hard and soft tissues may occur as a consequence of child's habit of placing foreign objects into the mouth. Foreign objects may become a potent source of pain and infection. The chance of these foreign objects getting impacted into the tooth is more when the pulp chamber is open either because of traumatic injury or a large carious exposure. Retrieval of foreign objects from the teeth in children is a challenging aspect of pediatric dental practice. These objects can be easily retrieved if they are located within the pulp chamber, but once the object has been pushed apically, their retrieval may be complicated. Apical surgical procedures may sometimes be necessary. In this paper, we present four interesting cases of unusual foreign bodies in the oral cavity. 

## 2. Case Report 1

**Figure 1:**
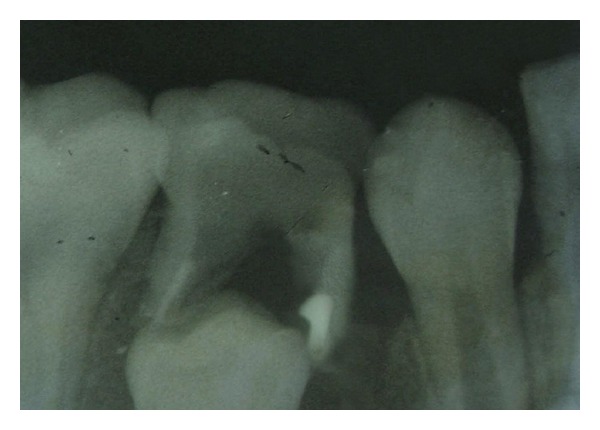
Intraoral periapical radiograph showing the radiopaque foreign body.

An 8-year-old boy presented to the Department of Pedodontics of Dr. HSJIDS with the chief complaint of swelling and pain in the lower right posterior region since 3-4 days. Intraoral examination revealed a fractured filling, grade 1 mobility of the tooth, tenderness on percussion, and abscess in relation to tooth 85. An intraoral periapical radiograph was advised; it revealed the presence of a sharp radiopaque material present in relation to the external surface of the mesial root (Figure 1).

**Figure 2:**
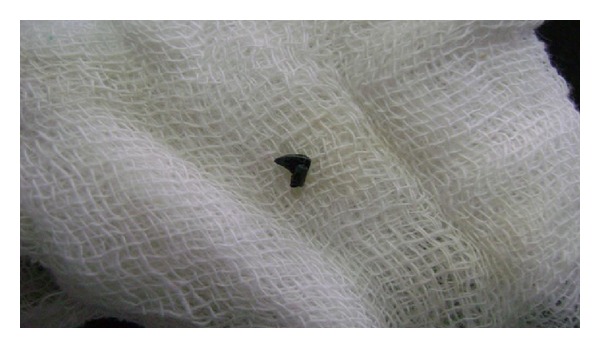
Removed tip of the metallic compass.

Extraction of the involved tooth was done, and the foreign object was removed that was found out to be a sharp tip of the metallic compass (Figure 2). On further questioning, it was told by the patient's parents that the child frequently used to use a metallic compass to take out the impacted food from the fractured filling.

## 3. Case Report 2

**Figure 3:**
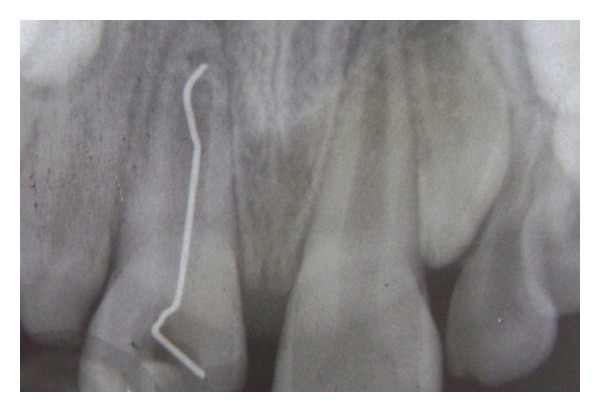
Intraoral periapical radiograph showing stapler pin in the pulp chamber of 11.

**Figure 4:**
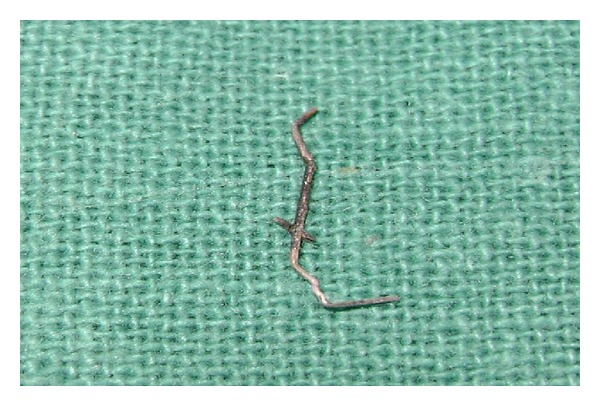
Removed stapler pin from the tooth.

**Figure 5:**
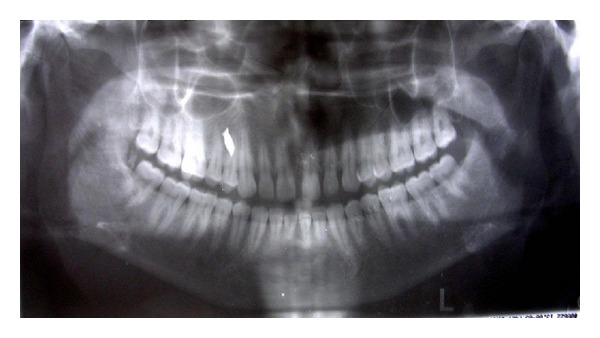
An OPG showing the copper strip.

An 11-year-old boy was brought to the Department of Pediatric Dentistry with the chief complaint of pain and swelling in the maxillary anterior region. He had suffered dental trauma a year back. Intraoral examination revealed fractured 11 with the slit-like opening involving the pulp chamber of tooth 11 (Figure 3). The tooth exhibited the following clinical features: swelling in the labial vestibule, grade 1 mobility, and tenderness on percussion. An intraoral periapical X-ray of the region was advised. The radiograph revealed a stapler pin in the pulp chamber of 11 (Figure 4). History revealed that the child had the habit of playing with the stapler; the pin got stuck in his tooth. Attempts by him to remove it were futile. The incident was concealed from his parents as he feared a reprimand or admonishment. A tetanus vaccine booster dose was administered to the patient in the very first appointment. The timing of presentation enabled us to solve the problem by removing of the stapler pin from the tooth (Figure 5) by making a conventional access cavity followed by copious irrigation of the pulp chamber to remove the debris present; routine endodontic procedure was followed by placement of dressing of nonsetting calcium hydroxide. Once the tooth was asymptomatic, it was obturated.

## 4. Case Report 3

**Figure 6:**
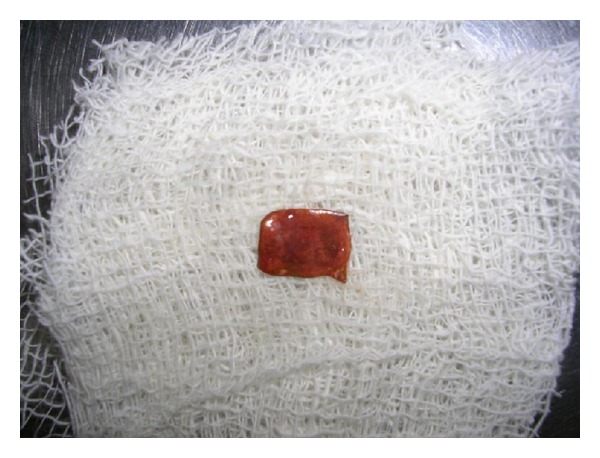
Removed copper strip.

An 28-year-old male patient reported to the Department of Oral Medicine and Radiology with a complaint of dull pain in upper right back region. The patient was an air conditioner mechanic and had met with an accident two months back; while repairing the air conditioner, it suddenly burst and he got severe injuries on his face and one of the fragments of the air conditioner got embedded in oral cavity. On examination, there was a fibrous swelling palpable in the vestibular area of the upper right premolars. An OPG radiograph was advised (Figure 5). The radiograph revealed a rectangular radiopacity in the premolar region. The area was explored under local anesthesia, and a rectangular copper strip approximately 0.7 × 1 cm was removed (Figure 6).

## 5. Case Report 4

**Figure 7:**
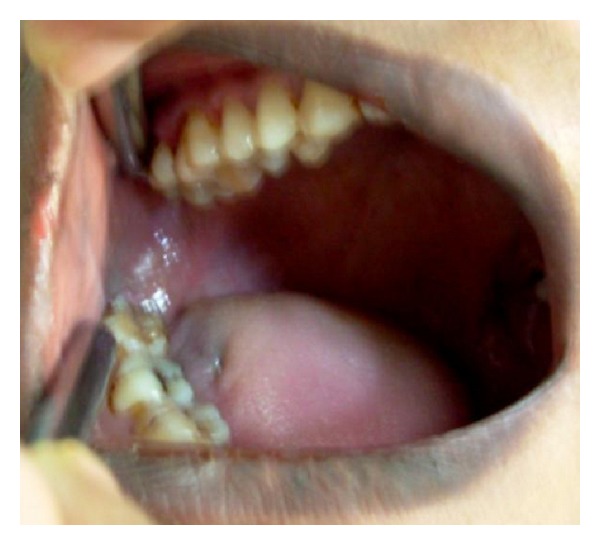
Intraoral view showing the broken needle.

**Figure 8:**
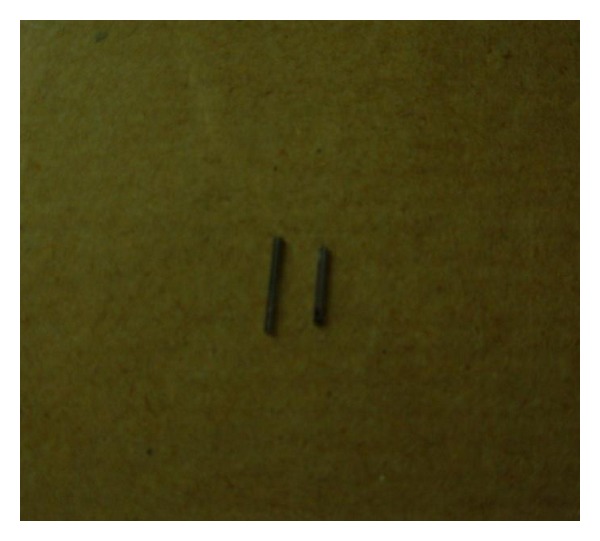
Fragments of the broken needle.

An 20-year-old male patient reported to the department with a history of breaking a sewing needle in upper right back area two days back while trying to remove the food debris (Figure 7). An IOPA radiograph was advised, and it revealed a radiopaque object that was the broken needle on both sides of 17. Under local anesthesia, the two fragments were removed (Figure 8).

## 6. Discussion

Self-inflicted oral injuries can be premeditated or accidental or can result from an uncommon habit. These injuries usually result from a foreign object or a patient's fingernail that habitually causes injury to the teeth or the gingival tissue. There are varying degrees of self-injurious behavior from simple fingernail biting to the extremes in self-mutilation [8–11]. The present case in which the mechanical trauma has been caused by the use of the metallic compass has not been reported till date to the best of authors' knowledge. These cases serve as another opportunity to emphasize the necessity of a comprehensive history which obtains the more subtle information relative to etiology. A proper case history and radiographic interpretation can lead to correct treatment plan.

Various foreign objects were reported to be lodged in the root canals and the pulp chamber, which ranged from pencil leads [12], darning needles [13], and metal screws [14] to beads [15] and stapler pins [16]. Grossman and Heaton [17] reported retrieval of indelible ink pencil tips, brads, a tooth pick, adsorbent points, and even a tomato seed from the root canals of anterior teeth left open for drainage. Toida et al. [18] has reported a plastic chopstick embedded in an unerupted supernumerary tooth in the premaxillary region of a 12-year-old Japanese boy.

Zillich and Picken [19] and Turner [20] cited cases wherein hat pins and dressmaker pins that were used to remove the food plugs from the root canals of maxillary and mandibular incisors undergoing endodontic treatment had eventually fractured inside the root canals of these teeth. Gelfma [21] and colleagues reported a case where a 3-year-old child had inserted two straws into the root canal of a primary central incisor, which was later extracted. Harris [22] reported the placement of varied objects within the root canals of maxillary anterior teeth. These included pins, wooden toothpick, a pencil tip, plastic objects, toothbrush bristles, and crayons. The patients had inserted these objects in the root canal to remove food plugs from the teeth. Placements of beads, a paper clip, and a stapler pin in the root canals of maxillary incisors were reported. Lamster and Barenie [23] reported insertion of a conical metallic object in the distal root of the primary left first molar.

A conventional practice employed during emergency root canal treatment involves leaving the pulp chamber open where pus continues to discharge through the canal and cannot be dried within a reasonable period of time. Weine [24] recommends that the patient remains in the office with a draining tooth for an hour or even more and finally ending the appointment by sealing the access cavity. With the access cavity closed, no new strains of microorganism system are introduced and food debris and foreign body lodgment within the tooth can be avoided [25].

A radiograph can be of diagnostic significance especially if the foreign body is radiopaque. Hunter and Taljanovic [6] summarized various radiographic methods to be followed to localize a radiopaque foreign object as parallax views, vertex occlusal views, triangulation techniques, stereo radiography and tomography. The visibility of different materials on plain radiographs depends on their ability to attenuate X-rays; foreign bodies may be visualized, depending on their inherent radiodensity and proximity with the tissue in which they are embedded [14]. Metallic objects, unless made of aluminum, are opaque on radiographs, as are most animal bones and all glass foreign bodies.

In patients who have had a penetrating injury, the nature of the foreign body determines the clinical behaviour; inert objects such as steel and glass may not cause significant inflammation to warrant their removal. Removal of organic foreign bodies is, however, mandatory, since these objects usually lead to secondary infection, with abscess and fistula formation. In our case, the various foreign bodies were the tip of the metallic compass, stapler pin, air conditioner copper chip, and fragments of broken needle that were removed timely and proper treatment plan could be carried out.
